# Treatment resistance NMDA receptor pathway polygenic score is associated with brain glutamate in schizophrenia

**DOI:** 10.1016/j.schres.2023.08.020

**Published:** 2023-10

**Authors:** Kira Griffiths, Sophie E. Smart, Gareth J. Barker, Bill Deakin, Stephen M. Lawrie, Shon Lewis, David J. Lythgoe, Antonio F. Pardiñas, Krishna Singh, Scott Semple, James T.R. Walters, Stephen R. Williams, Alice Egerton, James H. MacCabe

**Affiliations:** a Department of Psychosis Studies, Institute of Psychiatry, Psychology & Neuroscience, King's College London, London SE5 8AF, UK; b NIHR Biomedical Research Centre at South London and Maudsley NHS Foundation Trust, London, UK; c MRC Centre for Neuropsychiatric Genetics and Genomics, Division of Psychological Medicine and Clinical Neurosciences, Cardiff University, Cardiff, UK; d Department of Neuroimaging, Institute of Psychiatry, Psychology & Neuroscience, King's College London, London SE5 8AF, UK; e Division of Neuroscience and Experimental Psychology, University of Manchester, M13 9PT, UK; f Division of Psychiatry, University of Edinburgh, Edinburgh, UK; g Division of Psychology and Mental Health, University of Manchester, M13 9PT, UK; h Greater Manchester Mental Health NHS Foundation Trust, Manchester M25 3BL, UK; i Cardiff University Brain Research Imaging Centre, Cardiff University, Cardiff CF24 4HQ, UK; j BHF Centre for Cardiovascular Science, University of Edinburgh, Edinburgh EH16 4TJ, UK; k Division of Informatics, Imaging and Data Sciences, University of Manchester, Manchester, UK

**Keywords:** Glutamate, schizophrenia, anterior cingulate cortex, magnetic resonance spectroscopy

## Abstract

Dysfunction of glutamate neurotransmission has been implicated in the pathophysiology of schizophrenia and may be particularly relevant in severe, treatment-resistant symptoms. The underlying mechanism may involve hypofunction of the NMDA receptor. We investigated whether schizophrenia-related pathway polygenic scores, composed of genetic variants within NMDA receptor encoding genes, are associated with cortical glutamate in schizophrenia. Anterior cingulate cortex (ACC) glutamate was measured in 70 participants across 4 research sites using Proton Magnetic Resonance Spectroscopy (^1^H-MRS). Two NMDA receptor gene sets were sourced from the Molecular Signatories Database and NMDA receptor pathway polygenic scores were constructed using PRSet. The NMDA receptor pathway polygenic scores were weighted by single nucleotide polymorphism (SNP) associations with treatment-resistant schizophrenia, and associations with ACC glutamate were tested. We then tested whether NMDA receptor pathway polygenic scores with SNPs weighted by associations with non-treatment-resistant schizophrenia were associated with ACC glutamate. A higher NMDA receptor complex pathway polygenic score was significantly associated with lower ACC glutamate (β = −0.25, 95 % CI = −0.49, −0.02, competitive p = 0.03). When SNPs were weighted by associations with non-treatment-resistant schizophrenia, there was no association between the NMDA receptor complex pathway polygenic score and ACC glutamate (β = 0.05, 95 % CI = −0.18, 0.27, competitive p = 0.79). These results provide initial evidence of an association between common genetic variation implicated in NMDA receptor function and ACC glutamate levels in schizophrenia. This association was specific to when the NMDA receptor complex pathway polygenic score was weighted by SNP associations with treatment-resistant schizophrenia.

## 1 Introduction

Schizophrenia is a heritable and polygenic disorder, with many common genetic variants, particularly single nucleotide polymorphisms (SNPs), contributing to overall risk of disease (Pardiñas et al., 2018; Purcell et al., 2009; Ripke et al., 2014; Trubetskoy et al., 2022). Schizophrenia-associated SNPs can be mapped to risk conferring genes (Trubetskoy et al., 2022), and many of these genes impact synaptic plasticity, the coding of post-synaptic protein complexes and glutamatergic neurotransmission (Ripke et al., 2014; Trubetskoy et al., 2022). Furthermore, some of the schizophrenia-associated genetic variation shows enrichment in genes which may be directly or indirectly involved in the functioning of the *N*-methyl-d-aspartate (NMDA) glutamate receptor subunit (Trubetskoy et al., 2022; Coyle et al., 2020). These genetic insights complement a body of preclinical and clinical evidence for a role of glutamate in schizophrenia aetiology (Howes et al., 2015; Olney and Farber, 1995; Moghaddam and Javitt, 2012). Alterations in brain glutamate levels in schizophrenia (Merritt et al., 2016; Merritt et al., 2021) may be heritable (Legind et al., 2019). However, confident associations between variability in brain glutamate levels and common genetic variation in schizophrenia have not been established.

Meta-analyses of ^1^H-MRS studies have detected elevations in brain glutamate in patients with schizophrenia compared to healthy volunteers (Merritt et al., 2016), although the results from individual studies may vary due to sample clinical characteristics, the brain region investigated and other methodological factors (Merritt et al., 2016; Merritt et al., 2021). A mega-analysis of patient level data found that glutamatergic metabolites in the anterior cingulate cortex (ACC) are positively associated with symptom severity (Merritt et al., 2021). Further, group comparisons suggest that ACC glutamatergic metabolites may be especially elevated in those who have shown a poor response to antipsychotic treatment and in treatment-resistant schizophrenia (Demjaha et al., 2014; Egerton et al., 2012, Egerton et al., 2018, Egerton et al., 2021; Iwata et al., 2019; Mouchlianitis et al., 2016; Tarumi et al., 2020). While glutamate concentrations measured with ^1^H-MRS reflect the total amount of MR-visible glutamate in a voxel rather than glutamate involved in neurotransmission specifically, increases in glutamate concentrations would be compatible with the NMDA receptor hypofunction model of schizophrenia (Olney and Farber, 1995; Olney et al., 1999; Gao and Snyder, 2013). This hypothesis proposes that hypofunction of NMDA receptors on GABAergic interneurons leads to a disinhibition of pyramidal neurons and increased cortical glutamate signalling (Olney et al., 1999; Gao and Snyder, 2013; Homayoun and Moghaddam, 2007; Lisman et al., 2008; Nakazawa et al., 2012; Pafundo et al., 2018). This explanation is supported by studies showing that administration of NMDA receptor antagonists such as ketamine and phencyclidine can elicit behavioural effects akin to the symptoms of schizophrenia in healthy controls (Anis et al., 1983; Krystal et al., 2003; Luby et al., 1959), and exacerbate symptoms in patients with schizophrenia (Javitt and Zukin, 1991). These compounds have also been reported to increase cortical glutamate concentrations (Moghaddam et al., 1997; Javitt et al., 2018; Rowland et al., 2005; Stone et al., 2012).

Evidence of altered glutamate in unaffected relatives of patients with schizophrenia (Legind et al., 2019; Keshavan et al., 2009; Lutkenhoff et al., 2010; Purdon et al., 2008; Thakkar et al., 2017; Tibbo et al., 2004) suggests that variations in brain glutamatergic metabolite levels may have a genetic basis. This is also supported by a study showing that the aggregate effect of three schizophrenia-associated SNPs within glutamate genes was positively correlated with Glx (glutamate + glutamine) in frontal cortical gray matter in young patients with schizophrenia (Bustillo et al., 2017). That study found no effect in the group of older patients or combined cohort (Bustillo et al., 2017), which in part may be due to the small effect size associations of individual SNPs.

Treatment-resistant schizophrenia has a distinct polygenic signal (Pardiñas et al., 2021). It is possible that this polygenic signal could also contribute to variation in ACC glutamate levels, as ACC glutamate may also differ according to antipsychotic response (Demjaha et al., 2014; Egerton et al., 2012, Egerton et al., 2018, Egerton et al., 2021; Iwata et al., 2019; Mouchlianitis et al., 2016). Polygenic scores can be composed of SNPs mapped to gene sets which reflect the encoding of different biological functions, termed pathway polygenic scores (Choi et al., 2023). The current study investigated associations between treatment resistance (TR) pathway polygenic scores, composed of SNPs within NMDA receptor encoding genes, and ACC glutamate in schizophrenia. Our primary hypothesis was that NMDA receptor pathway polygenic scores, weighted according to SNP associations with treatment resistance, would be positively associated with ACC glutamate levels.

## 2 Methods

### 2.1 Ethics

The study had NHS Research Ethics Committee (15/LO/0038) approvals and all participants provided written informed consent.

### 2.2 Participants

This patient group is a subsample of the STRATA-1 imaging cohort presented in a previous publication (Egerton et al., 2021). Participants included in this study provided blood samples for genotyping and completed an MRI scan with ^1^H-MRS data.

Patients were recruited across four research sites: Cardiff University, University of Edinburgh, University of Manchester and King's College London. Study inclusion required participants to be aged between 18 and 65, meet Diagnostic and Statistical Manual of Mental Disorders (DSM-5) criteria for schizophrenia or schizophreniform disorder and be able to understand and consent to study procedures, including a sufficient level of English. Exclusion criteria were poor medication adherence (defined as a score of <3 on the Compliance Rating Scale [CRS]) (Kemp et al., 1996), current pregnancy, previous severe head injury involving loss of consciousness for >5 min, currently meeting International Classification of Diseases (ICD) criteria for harmful substance misuse or psychotic disorder secondary to substance misuse and any Magnetic Resonance imaging (MRI) contraindications, such as implanted electronic devices or metallic objects. Participants were also excluded if they have received treatment with clozapine in the last 3 months prior to study screening given potential effects of clozapine on brain glutamate concentrations (McQueen et al., 2021).

### 2.3 Defining good and poor antipsychotic response

Good and poor antipsychotic responders were recruited based on a priori criteria of symptom severity above or below a certain threshold. The treatment history and current symptom severity of potential participants were assessed through structured interview and review of medical records. Antipsychotic response was defined as 1) having treatment with only one antipsychotic since illness onset, or if there were any treatment changes then these were due to adverse effects rather than non-response; 2) Clinical Global Impression-Schizophrenia Scale (CGI-SCH) severity score < 4; 3) Positive and Negative Syndrome Scale (PANSS) total score < 60. Antipsychotic non-response was defined as 1) documented treatment with at least two antipsychotics for >4 weeks, at doses above the minimum therapeutic doses as defined by the British National Formulary; 2) a CGI-SCH score > 3; 3) PANSS total score at least 70.

The study included an initial screening period in which antipsychotic response status was determined. Recruitment aimed for a 1:1 ratio of antipsychotic responders and non-responders and therefore participant enrolment a given group stopped when study site recruitment targets were reached.

### 2.4 ^1^H-MRS and quality control procedures

Metabolite concentrations were measured using ^1^H-MRS at 3 Tesla according to the protocol described by Egerton et al. (2021). Spectra were analysed in LCModel version 6.3.1L using a standard LCModel basis set. Glutamate concentration estimates were water-referenced and corrected for voxel tissue content (Egerton et al., 2021), to give the primary outcome measure of Glu_corr_. For completeness, we also report results from analyses using Glx concentration estimates water-referenced and corrected for voxel tissue content (Glx_corr_) as the outcome_._ The spectral line width and signal-to-noise ratio were reviewed as part of quality control procedures (Egerton et al., 2021). Spectra were excluded if 1) the line width was 2 standard deviations above the overall mean for the voxel across all participants at all sites, or 2) the signal-to-noise ratio was 2 standard deviations below the overall mean for the voxel across all participants at all sites. Individual metabolite concentrations were excluded if the Cramér-Rao lower bounds value was 20 % or higher. There were no significant group differences in the signal-to-noise ratio, full width half maximum or Cramér-Rao lower bounds values between good and poor antipsychotic responders (Egerton et al., 2021). Given that different scanners were used across the four research sites, all metabolite concentrations were converted to z-scores to account for site effects (see Egerton et al., 2021 supplementary materials for a detailed discussion of scanner site effects).

### 2.5 Genetics

#### 2.5.1 Genotyping and quality control procedures

Full details of the STRATA-1 genetics pipeline are outlined in the supplementary methods. Samples were genotyped at the MRC Centre for Neuropsychiatry Genetics and Genomics in CU, using the HumanOmniExpress-24 v1.2 BeadChip. Quality control procedures were implemented using PLINK software (version 1.9) (Chang et al., 2015; Purcell et al., 2007) following standard protocols (Anderson et al., 2010) (see supplementary methods). Genotypes were then imputed using the Michigan Imputation Server (Das et al., 2016) with the reference panel ancestry set to mixed. Imputed genotype data was preserved in dosage format. SNPs which deviated from the Hardy-Weinberg equilibrium (HWE) at p-mid < 1e−6, had an imputation quality score (INFO) < 0.9, or a minor allele frequency (MAF) < 0.01 were removed.

#### 2.5.2 NMDA gene sets for TR pathway polygenic scores

Gene sets from the Gene Ontology (GO) collection (Ashburner et al., 2000) were obtained from the open access Molecular Signatures Database (MSigDB) (v7.2; https://www.gsea-msigdb.org/gsea/msigdb/index.jsp). All available GO gene sets on the MSigDB which were tagged with term ‘NMDA’ were downloaded (supplementary materials, Appendix I), because NMDA receptor function is hypothesised to underlie aberrant cortical glutamate signalling in schizophrenia (Olney and Farber, 1995). Importantly, the MSigDB does not include all current GO gene sets which are tagged by the term ‘NMDA’ because many of these gene sets overlap. As part of the database curation, MSigDB minimises these redundancies by computing the Jaccard coefficient between each pair of gene sets. A pair is marked as highly similar if the Jaccard coefficient exceeds 0.85, and these highly similar sets are clustered together. Overall, our search of the MSigDB returned two GO gene sets which were tagged with the term ‘NMDA’. These gene sets were: 1) the NMDA selective glutamate receptor complex and 2) positive regulation of NMDA glutamate receptor activity (see Appendix I for list of genes included in each gene set).

#### 2.5.3 Generating TR NMDA receptor pathway polygenic scores

TR NMDA receptor pathway polygenic scores were constructed using PRSet (Choi et al., 2023) implemented in PRSice-2 software (Choi and O'Reilly, 2019). Each individual had two polygenic scores; one with SNPs listed in the NMDA selective glutamate receptor complex gene set and the other with SNPs listed in the positive regulation of NMDA glutamate receptor activie gene set (Appendix I). Only SNPs within introns and exons defined by the human assembly GRCh37-hg19 gene boundaries were included. SNPs were weighted by their association with treatment-resistant schizophrenia. Here, we used summary statistics provided by the treatment resistance GWAS (TRS GWAS) (Pardiñas et al., 2021; downloadable from https://walters.psycm.cf.ac.uk/), which compared genome-wide variation of schizophrenia cases with and without a diagnosis of treatment resistance (see supplementary methods). The TRS GWAS did not include any individuals from the STRATA-1 cohort. All overlapping SNPs in the discovery TRS GWAS and target STRATA-1 cohort were included in the TR NMDA receptor pathway polygenic scores (i.e. p-value threshold = 1) because pathway polygenic scores containing a small portion of SNPs may be unrepresentative of the whole gene set (Choi et al., 2023).

### 2.6 Statistical analysis

#### 2.6.1 TR NMDA receptor pathway polygenic scores and ACC glutamatergic metabolites

Linear regression models were used to test associations between the two TR NMDA receptor pathway polygenic scores and ACC Glu_corr_. These TR NMDA receptor pathway polygenic scores were named 1) the NMDA selective glutamate receptor complex polygenic score (NMDAR-PGS) and 2) the positive regulation of NMDA glutamate receptor activity polygenic score (NMDAR-PGS-2). Included SNPs were weighted by their association with treatment-resistant schizophrenia compared to treatment responsive schizophrenia (TRS GWAS) (Pardiñas et al., 2021). The first model included the NMDAR-PGS as the independent variable of interest and ACC Glu_corr_ as the outcome. The second model included the NMDAR-PGS-2 as the independent variable of interest and ACC Glu_corr_ as the outcome. Both models were adjusted for age, sex and chlorpromazine equivalent (CPZE) dose given their relationship with brain glutamate (Merritt et al., 2021; Egerton et al., 2021; Marsman et al., 2013; Sailasuta et al., 2008). Antipsychotic type and the number of previous antipsychotic trails were not included in models because these variables were not significantly associated with ACC Glu_corr_. or Glx_corr_. To adjust for population structure, the first five genetic principal components, plus any associated with the outcome at p < 0.05, were also included as covariates (Tucker et al., 2014). We also included antipsychotic response group as a covariate as ACC Glu_corr_ was elevated in poor compared to good antipsychotic responders (Egerton et al., 2021). Antipsychotic response group was a binary variable defined by the a priori recruitment criteria. For completeness, we also report results from analyses using Glx_corr_ as the outcome.

For both TR NMDA receptor pathway polygenic scores, raw and competitive p-values were provided by PRSet. The raw p-value tests the association between each TR NMDA receptor pathway polygenic score and ACC Glu_corr_, whereas the competitive p-value tests whether each TR NMDA receptor pathway polygenic score is more strongly associated with ACC Glu_corr_ than a polygenic score generated from a background gene set of the same size. Here, competitive p-values were obtained by comparing the observed TR NMDA receptor pathway polygenic score association with the 10,000 permuted null p-value distribution of background gene set polygenic scores (Choi et al., 2023). We examined the viability of three background gene sets for use in competitive p-value calculation. To do this, we obtained background gene sets including all 1) protein-coding, 2) brain expressed, or 3) synaptic genes (see supplementary methods: background gene set curation). We then tested associations between ACC Glu_corr_ and the polygenic signal from each background gene set (see supplementary methods, supplementary Fig. 1). Given that the most variance in ACC glutamate was explained by a polygenic score including SNPs mapped to synaptic genes (supplementary Table 1), we used the synaptic gene set as our background gene set when generating competitive p-values in the main TR NMDA receptor pathway polygenic score analyses.

#### 2.6.2 Exploratory analysis

In the primary analysis, the SNPs in the two TR NMDA pathway polygenic scores were weighted by their association with treatment-resistant schizophrenia compared to treatment responsive schizophrenia (TRS GWAS) (Pardiñas et al., 2021). As an exploratory analysis, we then tested for associations between ACC glutamate and NMDA receptor pathway polygenic scores including SNPs weighted by other training sets analysed by Pardiñas et al. (2021) namely i) non-treatment-resistant schizophrenia versus healthy controls (“PGC GWAS”) and ii) treatment-resistant schizophrenia versus healthy controls (“CLOZUK GWAS”). Given the high genetic correlation of treatment responsive and treatment-resistant schizophrenia (Pardiñas et al., 2021), this analysis was performed to provide a more nuanced understanding of the relative contribution(s) of the shared and distinct genetic variation between these disorders to ACC glutamate. Descriptions of these GWAS cohorts are provided in the supplementary methods.

## 3 Results

Samples from 89 participants passed genotype quality control procedures and were available for polygenic score analyses. Of those, 70 participants also had ACC Glu_corr_ or ACC Glx_corr_ data. Clinical and demographic characteristics of the sample are reported in Table 1.

**Table 1:** Sample characteristics of participants with genotyped and ^1^H-MRS glutamate data which passed quality control (n = 70).

Variable	Value
Antipsychotic responder/non-responder	36/34
Sex male/female	57/13
Age (years)	29.71 ± 8.96
Age of onset (years)	24.77 ± 6.70
Duration of illness (years)	4.9 ± 6.70
Diagnosis psychosis unspecified/schizophrenia/delusional disorder	18/51/1
CPZE dose (mg/day)	396.07 ± 316.81
Benzodiazepine yes/no	8/62
Antidepressant yes/no	13/57
Current smoking no/less than daily/daily	28/3/39
Current cannabis yes/no	54/16
Self-reported ethnicity	
White	37
Black	18
Mixed White Black	5
Asian	5
Other	5
Antipsychotic	
Amisulpride	6
Aripiprazole	16
Olanzapine	14
Quetiapine	6
Risperidone	13
Other^a^	15
Symptom severity	
PANSS total	69.23 ± 17.74
PANSS positive	17.14 ± 2.24
PANSS negative	17.13 ± 5.36
PANSS general	34.96 ± 8.72
Glutamatergic metabolites	
ACC Glu_corr_	0.02 ± 0.99
ACC Glx_corr_	0.02 ± 1.03

Values are expressed as mean ± SD unless otherwise specified. CPZE = chlorpromazine equivalent antipsychotic dose. ACC Glu_corr_ = anterior cingulate cortex glutamate corrected for voxel tissue content and z-scored. ACC Glx_corr_ = anterior cingulate cortex glutamate corrected for voxel tissue content and z-scored. Clinical and demographic characteristics of the STRATA-1 cohort stratified by antipsychotic response are reported in Egerton et al. (2021).

a Antipsychotics included in “other” were prescribed to five participants or fewer and include: clopixol, paliperidone, haloperidol, flupenthixol, amisulpride & aripiprazole combination, quetiapine & amisulpride combination, olanzapine & paliperidone combination, and zuclopenthixol.

### 3.1 TR NMDA receptor pathway polygenic scores and ACC glutamatergic metabolites

**Fig. 1:**
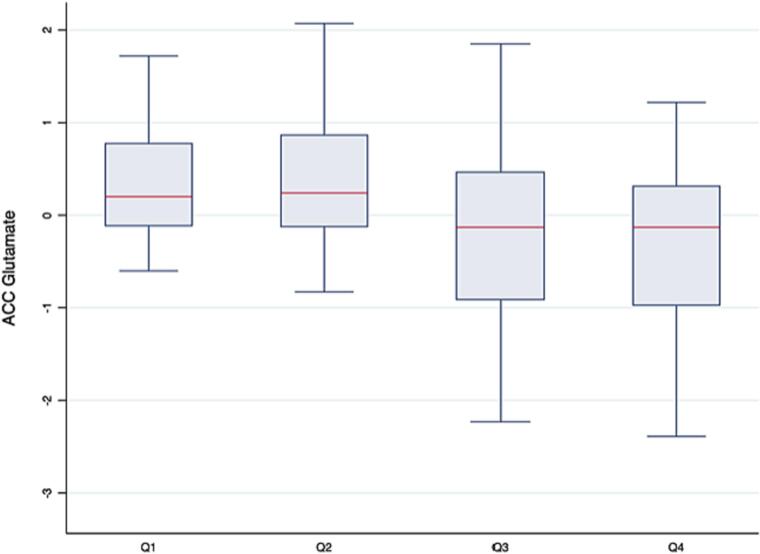
Graph showing box and whisker plots of anterior cingulate cortex glutamate (ACC Glu_corr_) across quartiles of the treatment resistance NMDA receptor complex pathway polygenic score (TR NMDAR-PGS). For each box plot, the median value of ACC glutamate is represented by the red line. Upper and lower values of ACC glutamate within each quartile are represented by error bars. (For interpretation of the references to color in this figure legend, the reader is referred to the web version of this article.)

The TR pathway polygenic score generated using the NMDA selective glutamate receptor complex gene set (TR NMDAR-PGS) was negatively associated with ACC Glu_corr_ after adjusting for age, sex, CPZE dose, antipsychotic response group and the first five genetic principal components (β = −0.25, 95 % CI = −0.49, −0.02, competitive p = 0.03) (Table 2, Fig. 1, and supplementary Table 2). The full regression model is reported in supplementary Table 3. Subsequent analyses confirmed that this main effect did not reflect collider bias from the inclusion of antipsychotic response as a covariate (Westreich and Greenland, 2013) (see supplementary results: supplementary Table 4). There was no significant association between the TR NMDAR-PGS and ACC Glx_corr_ (supplementary Table 2). The TR pathway polygenic score generated using the positive regulation of NMDA glutamate receptor activity gene set was not significantly associated with ACC Glu_corr_ or Glx_corr_ (supplementary Table 2).

**Table 2:** Associations between NMDAR-PGS and glutamatergic metabolite concentrations in the anterior cingulate cortex.

Discovery GWAS for SNP weights	No. SNPs	Glutamate			Glx		
		β	95 % CI	p-Value	β	95 % CI	p-Value
TRS	83	−0.25	−0.49, −0.02	0.03	−0.09	−0.33, 0.16	0.64
PGC	88	0.05	−0.18, 0.27	0.79	0.08	−0.15, 0.31	0.56
CLOZUK	87	−0.22	−0.49, 0.05	0.09	−0.09	−0.37, 0.19	0.40

The primary analysis concerned NMDA receptor pathway polygenic scores weighted by SNP effect size associations provided by the TRS GWAS. In an exploratory analysis, SNPs included in the NMDA receptor pathway polygenic scores were weighted according to their effect size associations reported in the i) PGC and ii) CLOZUK GWAS. β is the coefficient for the association between the NMDA receptor complex pathway polygenic score (NMDAR-PGS) and anterior cingulate cortex glutamate or Glx. Metabolite concentrations were corrected for voxel tissue content and standardised. p-Value = competitive p-value. Competitive p-values were calculated by PRSet using synaptic expressed genes as the background gene set.

### 3.2 Sensitivity analysis

Because polygenic scores may differ by ancestry (Duncan et al., 2015) and an association between poor clinical outcomes in schizophrenia and ethnicity has been reported (Morgan et al., 2017), we performed a post-hoc analysis to understand the potential impact of ethnicity on the association between the TR NMDA-PGS and ACC Glu_corr_ in our cohort. A sensitivity analysis investigating the association between the TR NMDAR-PGS and ACC Glu_corr_ was performed on the majority group for self-reported ethnicity (white participants: 53 %). The association between the TR NMDAR-PGS and ACC Glu_corr_ was not significant when analysis was restricted to white participants (β = −0.22, 95 % CI = −0.61, 0.17, competitive p = 0.25), although the effect size was similar to the results from the total group analysis. Additionally, analysis of variance found no significant difference in the TR NMDA-PGS between ethnicity groups (p = 0.42).

### 3.3 Exploratory analysis

When SNPs included in the TR NMDAR-PGS were weighted by effect sizes reported in the PGC and CLOZUK GWAS, there were no significant associations between the NMDAR-PGS and ACC Glu_corr_ or Glx_corr_ (Table 2).

## 4 Discussion

This study investigated associations between NMDA receptor pathway polygenic scores and ACC glutamate levels in schizophrenia. We found a significant association between the NMDA receptor complex pathway polygenic score (NMDAR-PGS) and ACC glutamate. Contrary to our primary hypothesis of a positive association, the direction of effect was negative. This relationship was only apparent when SNPs included in the NMDAR-PGS were weighted by their association with treatment-resistant schizophrenia compared to non-treatment-resistant schizophrenia. No significant associations between the NMDAR-PGS and ACC Glu_corr_ were found when SNPs were weighted by their associations with i) treatment-resistant schizophrenia versus healthy controls or ii) non-treatment-resistant schizophrenia versus healthy controls. These results suggest that genetic variation within NMDA receptor encoding genes that is associated with treatment resistance in schizophrenia may contribute to ACC glutamate levels in this disorder, and further implicate NMDA receptor function as contributing to variation in brain glutamate levels as measured in vivo with ^1^H-MRS.

One previous study has assessed the relationship between schizophrenia-associated genetic variation and brain measures of glutamate in schizophrenia. That study found an association between a glutamate genetic risk score, comprised of three SNPs identified in the Psychiatric Genetics Consortium Schizophrenia GWAS, and gray matter Glx in young schizophrenia patients (Ripke et al., 2014; Bustillo et al., 2017). Our approach of using pathway polygenic scores, as opposed to studying the effects of few SNPs in isolation, has the advantage of increased power (Dudbridge, 2013) while maintaining information on individual-level variation within different functional pathways across the genome (Choi et al., 2023). Further, we primarily focused on genetic variation associated with a specific subgroup of patients with schizophrenia (treatment-resistant schizophrenia) (Pardiñas et al., 2021) because aberrant ACC glutamate may be particularly relevant in the pathophysiology of poor antipsychotic response (Gillespie et al., 2017). However, the relatively smaller sample size of the treatment-resistant schizophrenia GWAS which was used to weight SNPs for our TR NMDAR-PGS should be highlighted, particularly when compared to the sample size of the wider PGC schizophrenia GWAS (Ripke et al., 2014; Trubetskoy et al., 2022). Within this context, the limited power of the TRS GWAS used in current study is an important limitation to note because this may have affected the accuracy of SNP weights for the TR NMDAR-PGS (Pardiñas et al., 2021).

The association between the TR NMDAR-PGS and ACC glutamate adds support to a role of NMDA receptor function in the aetiology of aberrant glutamatergic signalling in schizophrenia. Genes coding for NMDA receptor function have been previously mapped onto behavioural phenotypes of schizophrenia using genetically engineered mouse models (Lee and Zhou, 2019). NMDA receptors consist of two obligatory NR1 subunits and two NR2 and/or NR3 subunits (Laurie and Seeburg, 1994). Mice with reduced expression of NR1 and knockout of NR2A/B subunits display behavioural abnormalities akin to those seen in PCP and ketamine models of schizophrenia, such as increased locomotion, impaired discrimination learning and stereotyped behaviour (Brigman et al., 2008; Duncan et al., 2004; Halene et al., 2009; Mohn et al., 1999; Sprengel et al., 1998). However, links between NMDA receptor genetic variation and brain glutamate are not well established. An association between NMDA receptor function and glutamate has been indirectly evidenced by studies demonstrating that the administration of NMDA receptor antagonists increases ACC glutamatergic metabolites (Javitt et al., 2018; Rowland et al., 2005; Stone et al., 2012; Napolitano et al., 2014). High dose administration of the NMDA receptor glycine site partial agonist D-cycloserine at high doses, which may have a net antagonistic effect at high doses (Emmett et al., 1991), has also been shown to increase Glx in the medial prefrontal cortex (Kantrowitz et al., 2016). Direct investigation of NMDA receptor function in schizophrenia has not been extensively studied given difficulties in developing radiotracers for these receptors (Kim et al., 2020; Knol et al., 2009). However, one molecular imaging study has reported a correlation between NMDA receptor dysfunction and negative symptoms in medicated patients (Pilowsky et al., 2006). Our results extend this literature to tentatively suggest that common genetic variation which is associated with an increased risk for treatment-resistant schizophrenia and located within NMDA receptor encoding genes may be associated with ACC glutamate.

As the TR NMDAR-PGS increased across the cohort we observed a reduction in ACC glutamate. Given that 1) a higher TR NMDAR-PGS represents more risk variants associated with treatment-resistant schizophrenia and 2) elevated glutamate is typically seen in cases of treatment resistance and poor antipsychotic response, the negative association observed in this study may be unexpected. Importantly, the functional interpretation of the current finding is limited by our understanding of the role of SNPs in overall gene function and the subsequent impact this may have on brain circuitry. For example, genetic variants within NMDA receptor genes have been associated with both increased and decreased potencies of glutamate and related metabolites (Yuan et al., 2015). A related point is that when creating pathway polygenic scores, PRSet only includes the treatment-resistant associated SNP with the largest GWAS effect size for each gene region. This means that SNPs in pathway polygenic scores may not have a causal effect on ACC glutamate but are in linkage disequilibrium with causal SNPs. Understanding mechanisms from gene pathways to biological phenotypes requires being able to disentangle these causal variants. This is a complex task, which requires large GWAS discovery samples and in some cases targeted sequencing approaches (Schaid et al., 2018). For now, we provide initial evidence for an association between SNPs that impact the NMDA receptor complex and brain glutamate levels as measured using ^1^H-MRS.

The polygenic profile of schizophrenia and treatment-resistant schizophrenia are highly correlated (Pardiñas et al., 2021). In using the TRS GWAS as the primary discovery dataset, SNPs included in the TR NMDAR-PGS were weighted by their effect size associations with treatment-resistant schizophrenia compared to treatment responsive schizophrenia. Our main finding therefore suggests that part of the polygenic signal which distinguishes treatment-resistant from non-treatment-resistant schizophrenia may be particularly relevant for variations in ACC glutamate. In support of this interpretation, exploratory analyses found no significant associations between the NMDA-PGS and ACC glutamate when SNP effect sizes were weighted by associations with treatment-resistant schizophrenia versus healthy controls (CLOZUK) or non-treatment resistant schizophrenia versus healthy controls (PGC). Overall, these results complement literature implicating altered ACC glutamate in cases of poor antipsychotic response and treatment-resistant illness (Demjaha et al., 2014; Egerton et al., 2013, 2018, 2021; Iwata et al., 2019; Mouchlianitis et al., 2016; Tarumi et al., 2020).

### 4.1 Strengths and limitations

We present glutamate imaging data from a large multi-centre and well-phenotyped representative cohort of schizophrenia patients. However, the sample size in the context of genetic studies is small and therefore a major limitation to this study. As such, results should be treated as preliminary and replication in larger samples is required. Notably, the power of polygenic scores increases with larger discovery GWAS and we used the largest available datasets of treatment-resistant schizophrenia and non-treatment resistant schizophrenia in analyses. However, our results and their interpretation will be limited by current knowledge of the biological function of gene sets, which are continually advanced and updated. There is also evidence that cortical metabolite concentrations may change with factors such as antipsychotic medication and age (Merritt et al., 2021), although we did control for these variables in analyses. As polygenic scores only summarise the effect of SNPs, we were unable to investigate the contribution of other types of genetic variation to the ^1^H-MRS glutamate signal, such as indels or copy number variants.

The discovery treatment resistant GWAS for the primary analysis and the discovery PGC and CLOZ GWAS for the exploratory analyses were samples of European ancestry individuals, whereas the ancestry of our cohort was more varied. The difference in ancestry composition between our sample and the treatment resistance GWAS is a limitation because polygenic scores can be less informative in populations more diverged from discovery GWAS cohorts (Gillespie et al., 2017). This is because GWAS summary statistics generated in one ancestry population may not capture other population-specific allele frequencies and linkage disequilibrium patterns. Novel polygenic scoring methods which integrate GWAS summary statistics from multiple populations can improve cross-population prediction (Ruan et al., 2022). Prioritisation of efforts to perform large TRS GWAS in non-European cohorts could therefore facilitate the development of polygenic scores with improved power in non-European populations. This is important to prevent exacerbation of health disparities in European ancestry vs. other underrepresented populations in clinical research. A final limitation is that we used self-reported ethnicity to describe the ancestry of the STRATA-1 sample rather than genetic measures of ancestry, which may better capture biological variation that is linked to population variability (James et al., 2021).

## 5 Conclusion

This study provides an initial indication of a link between treatment-resistant schizophrenia-associated common genetic variation within NMDA receptor encoding genes and glutamate in schizophrenia. If replicated, a potential implication of this research could be the application of pathway polygenic scores in identifying those at relatively more or less risk of glutamatergic pathophysiology.

## Funding

STRATA is funded by a grant from the 10.13039/501100000265Medical Research Council (MRC) to JHM, grant reference MR/L011794. KG is in receipt of a PhD studentship from the 10.13039/501100000272National Institute for Health Research (NIHR) 10.13039/100014461Biomedical Research Centre at 10.13039/100009362South London and Maudsley (SLaM) NHS Foundation Trust and 10.13039/501100000764King's College London. JHM and AE are part funded by the NIHR 10.13039/100014461Biomedical Research Centre at SLaM. AFP is supported by an 10.13039/501100000691Academy of Medical Sciences “Springboard” award (SBF005\1083). The views expressed are those of the authors and not necessarily those of the NHS, the BRC, the NIHR or the Department of Health.

## CRediT authorship contribution statement

KG, AE and JHM were responsible for the overall research idea and scientific interpretation of the data. KG performed the analyses and wrote the manuscript. SES, AFP and JTRW contributed to scientific interpretation of the data. JHM, AE, GJB, BD, SML, SL, DJL, KS, SS, JTRW, SRW contributed to the design and implementation of the STRATA-1 study. All authors contributed to editing of the manuscript and approved the final version.

## Declaration of competing interest

JTW is an investigator on a grant from Takeda Pharmaceuticals Ltd. to Cardiff University, for a project unrelated to the work presented here. SES is employed on this grant. GJB receives honoraria for teaching from GE Healthcare. All other authors have no conflicts of interest to disclose.

## Data Availability

At the time of submission, the data governance frameworks are being put in place to make a fully anonymized version of the data available to the wider research community via the TranSMART data sharing platform: https://transmartfoundation.org/. To apply for access to the data, please contact JHM at james.maccabe@kcl.ac.uk.

## Appendix A Supplementary data

Supplementary material Image 1
