# Supplementary material for: Treatment resistance NMDA receptor pathway polygenic score is associated with brain glutamate in schizophrenia
Source: Schizophr Res. 2023 Oct;260:152–9. doi: 10.1016/j.schres.2023.08.020 (PMC10873209; doi:10.1016/j.schres.2023.08.020)
Supplement: Supplementary file 1 — Supplementary material [file mmc1.docx]

Supplementary Material

# Supplementary Methods

- 1. **STRATA-1 Genotyping and quality control procedures**
     1. **Blood samples for genetic analysis**

For the STRATA-1 genetics cohort, venous blood samples were drawn from 91 individuals and stored at the research collection site, before transfer to the MRC Centre for Neuropsychiatry Genetics and Genomics, Cardiff University. Samples were genotyped using HumanOmniExpress-24 v1.2 BeadChip where genomic positions of variants were labelled according to the human assembly GRCh37 (hg19). Genotyped data files were then transferred to King’s College London for quality control procedures, imputation and subsequent analyses.

- - 1. **Basic quality control**

A total of 91 samples genotyped at 712,027 markers were available. PLINK version 1.9 (Chang et al., 2015; Purcell et al., 2007) was used to implement pre-imputation quality control procedures on the genotyped data following standard protocol (Anderson et al., 2010). Individuals were excluded from the sample if estimates of the inbreeding coefficient F (Hill., 1996) indicated ambiguous sex (F = 0.2 - 0.8) or there was a discrepancy between the genotypic and self-reported sex. Any discrepancies were checked with the data collection site to confirm no errors were made during manual entry of phenotypic data. Samples with a genotype call < 95%, or high pairwise relatedness (pi-hat > 0.1) were also removed. Single nucleotide polymorphisms (SNPs) were excluded if the minor allele frequency (MAF) was less than 0.01, if SNPs deviated from Hardy-Weinberg Equilibrium (HWE) at p-mid < 1e-6, or the minimum marker call rate was < 95%.

Of the 91 genotyped samples, two were removed due to sex inconsistencies in the comparison of X-chromosome heterozygosity and self-reported gender. Out of the 713,017 genotyped markers, 4,743 (0.67%) did not pass basic quality control filters. The curated dataset for subsequent imputation contained 89 individuals (73 males) genotyped at 708,284 markers.

- - 1. **Imputation**

Genome harmoniser was implemented to align all variants to the forward strand and 15,058 variants were removed at this stage (15,055 SNPs excluded during realignment and 3 SNPS were duplicated). Genotype imputation was performed using the Michigan Imputation Server (Das et al., 2016) https://imputationserver.sph.umich.edu). Data uploaded to the Michigan Imputation Server underwent in-house quality control before being passed through the imputation pipeline (see: https://imputationserver.readthedocs.io/en/latest/pipeline/). Data passing in-house quality control were phased using Eagle v2.4 (Loh et al., 2016) and imputed using minimac4 algorithm with the Haplotype Reference Consortium (HRCr1.1) reference panel (GRCh37/hg19 array build) with population set to mixed. Sex chromosomes were not imputed given the current lack of consensus on methods to analyse autosomal genetics in GWAS and subsequent polygenic score models (Choi, Mak and O’Reilly., 2020).

- - 1. **Post imputation quality control**

The quality of imputation for each missing genotype was assessed using the R^2^ imputation quality metric (INFO) in the chr*.info files provided by the Michigan Imputation Server. The average R^2^ in our sample across the whole genome was 0.4, suggesting adequate imputation quality for our sample (Stanaway et al., 2019). SNP names and coordinates were updated to the 1000 Genomes Reference Panel to match the treatment resistance GWAS (Pardiñas et al., 2021). Post-imputation filters were then applied (INFO score < 0.9, minor allele frequency < 0.10, deviation from Hardy-Weinberg equilibrium at p-mid < 1e-6), and the final curated dataset contained 89 samples, genotyped at 3,901,405 SNPs. Genotypes were preserved in dosage format to account for uncertainty in the imputed genotypes.

- 1. **Discovery GWAS datasets for polygenic score construction**

Our primary analysis used NMDA receptor pathway polygenic scores containing SNPs weighted by their effect size in the treatment resistance GWAS (Pardiñas et al., 2021). In that GWAS, the distinct genetic architecture of treatment-resistant schizophrenia was examined by estimating effect size differences between schizophrenia samples with and without treatment resistance (Pardiñas et al., 2021). To achieve this, Pardiñas and colleagues combined data from two independent GWAS: CLOZUK (a treatment-resistant schizophrenia sample) and PGC (a modified version of the PGC-2 sample to remove any cases of treatment resistance, leaving a schizophrenia sample who were considered responsive to antipsychotic treatment). Combined data from the CLOZUK and PGC cohorts gave a dataset of 83,841 participants for the treatment resistance GWAS (21,264 ‘treatment responsive’ schizophrenia cases, 10,501 treatment-resistant schizophrenia cases and 52,076 healthy controls).

Summary statistics from the treatment resistance GWAS are reported in a similar format to summary statistics from other GWAS performed in a more conventional manner. In brief, a SNP association with a positive direction of effect represents a variant more commonly found in cases of treatment-resistant schizophrenia compared to treatment responsive schizophrenia. When polygenic scores were generated using the weighted effect sizes of SNPs from the treatment resistance GWAS, the treatment resistance polygenic score was positively associated with treatment-resistant schizophrenia in two independent replication samples (samples comparing treatment-resistant schizophrenia and non-treatment resistant schizophrenia cases).

Collation of the CLOZUK sample is described in detail by Pardiñas and colleagues (Pardiñas et al., 2018). Samples include those with a clinician reported diagnosis of treatment-resistant schizophrenia, such that clozapine was prescribed after the failure of two antipsychotic treatment trials of adequate dosage and duration. The schizophrenia sample with no diagnosis of treatment resistance was collated from a modified PGC-2 dataset. This procedure is described in Pardiñas et al. (Pardiñas et al., 2021). Here, all cases with a diagnosis of treatment-resistant schizophrenia were removed. Because treatment resistance is often underreported, this final modified PGC schizophrenia sample will contain some individuals who do not respond to non-clozapine antipsychotic treatment. However, it is expected that these individuals will comprise a minority of the overall sample.

- 1. **Background gene set curation**

PRSet calculates competitive p-values, which test whether the NMDA receptor pathway polygenic score is more strongly associated with the outcome (ACC Glu_corr_) than a pathway polygenic score containing SNPs within gene regions randomly selected from a background gene set. We examined the viability of three background gene sets for use in competitive p-value calculation. These background gene sets were constructed of either 1) all protein-coding, 2) all brain expressed, or 3) all synaptic genes. When sourcing these background gene sets, we only included autosomal genes (chromosomes 1-22), because subsequent polygenic analyses also only included autosomal SNPs. All gene positions were mapped to the human genome build 37 (GRCh37) coordinates to correspond with genomic positions in the STRATA-1 dataset.

Protein-coding genes (n = 20,082) were extracted from annotation files sourced from the GENCODE release 38 (https://www.gencodegenes.org/human/release_38lift37.html). Genes with an elevated expression in brain tissue (brain expressed genes; n = 2,441) were downloaded from the Human Protein Brain Atlas (Thul and Lindskog., 2018) (https://www.proteinatlas.org/humanproteome/brain/human+brain). Synaptic genes (n = 1233) were downloaded from the SynGo database v1.1 (Koopmans et al., 2019) (https://www.syngoportal.org/help.html). We compared the viability of these three background gene sets for use in competitive p-value calculation for our primary analysis. To do this, we tested associations between anterior cingulate cortex glutamate (ACC Glu_corr_) and background pathway polygenic scores. Using PLINK software, SNPs in the STRATA-1 genotyped dataset were retained if they fell within gene regions defined by gene annotations for the three background gene sets. PRSet was used to generate three background gene set polygenic scores, which included SNPs mapped to 1) protein-coding, 2) brain expressed, or 3) synaptic genes. SNPs were weighted by their association with treatment-resistant schizophrenia and a p-value threshold of 1 was used. Background gene set pathway polygenic scores were standardised and tested for an association with ACC Glu_corr_, controlling for age, sex, chlorpromazine equivalent antipsychotic dose, antipsychotic response status and the first 5 genetic principal components. All associations between background gene set pathway polygenic scores and ACC Glu_corr_ were not significant (supplementary Figure 1; supplementary Table 1).

# Supplementary Results

**2.1. Selection of background gene set**


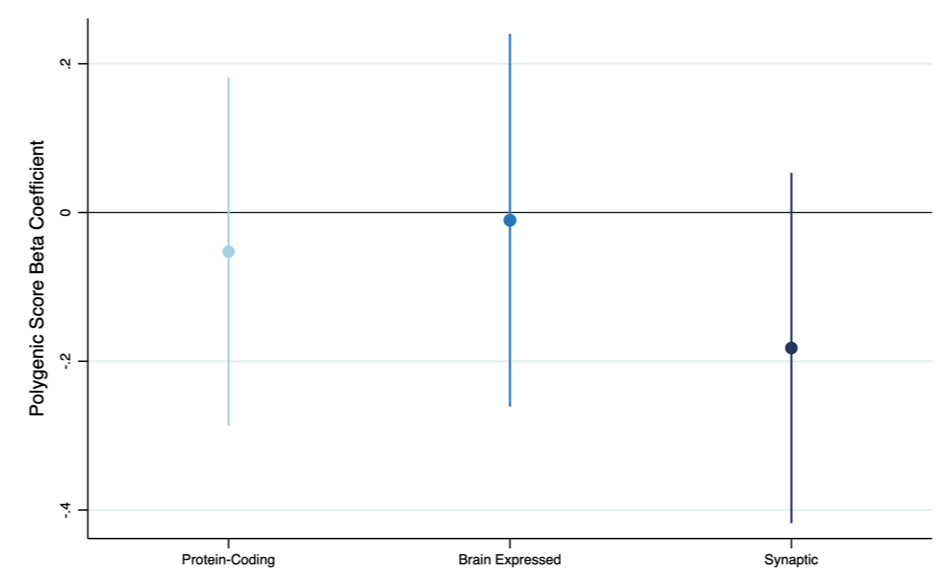


**Supplementary Figure 1.** Beta coefficients and 95% confidence intervals from regression analyses testing associations between treatment resistance polygenic scores and ACC glutamate. In each model, polygenic scores (including SNPs mapped to protein coding, brain expressed or synaptic genes) were the independent variable and anterior cingulate cortex glutamate corrected for voxel tissue content and z-scored was the outcome of interest. SNPs included in polygenic scores are weighted by their effect size association with treatment resistant schizophrenia (p-value threshold = 1). All models are adjusted for age, sex, chlorpromazine equivalent dose, antipsychotic response status and the first five genetic principal components.

| **TRS Polygenic Score** | **No. SNPs** | **PRS R^2^** | **Full R^2^** | **β** | **95% CI** | **p** |
| --- | --- | --- | --- | --- | --- | --- |
| ^1^Protein coding | 40,286 | 0.009 | 0.291 | -0.05 | -0.29, 0.18 | 0.65 |
| ^2^Brain expressed | 12,196 | 0.017 | 0.288 | -0.01 | -0.26, 0.24 | 0.93 |
| ^3^Synaptic | 5,938 | 0.089 | 0.317 | -0.18 | -0.42, 0.05 | 0.13 |

**Supplementary Table 1.** Output from linear regression models testing associations between treatment resistance polygenic scores and anterior cingulate cortex glutamate

TRS = treatment resistant schizophrenia.^1^SNPs included within gene regions of all protein coding genes; ^2^SNPs included within gene regions of all brain expressed genes; ^3^SNPs included within gene regions of all synaptic genes. PRS R^2^ = variance explained by the polygenic score; Full R^2^ = variance explained by the full model, including all covariates. All models are adjusted for age, sex, chlorpromazine equivalent dose, antipsychotic response status and the first 5 genetic principal components.

**2.2. Supplementary results – primary analysis**

**Supplementary Table 2.** PRSet results for associations between NMDA receptor pathway polygenic scores and anterior cingulate cortex glutamatergic metabolites

|  |  |  | Glutamate | | | | | Glx | | | | |
| --- | --- | --- | --- | --- | --- | --- | --- | --- | --- | --- | --- | --- |
| Gene Set | No. genes | No. SNPs | PRS R^2^ | β | 95% CI | Raw p | p-value | PRS R^2^ | β | 95% CI | Raw p | p-value |
| GOBP Positive Regulation of NMDA Glutamate Receptor Activity | 6 | 18 | 1.93E-05 | -0.04 | -0.33, 0.26 | 0.97 | 0.97 | 0.00 | 0.06 | -0.24, 0.36 | 0.61 | 0.61 |
| GOCC NMDA Selective Glutamate Receptor Complex | 10 | 83 | 0.06 | -0.25 | -0.49, 0.02 | 0.04 | 0.03* | 0.01 | -0.09 | -0.33, 0.16 | 0.37 | 0.37 |

*p < 0.05

All polygenic scores were calculated at a p-value threshold of 1 to maximise the number of SNPs included for each gene set. SNPs were weighted by their association with treatment resistant schizophrenia versus treatment responsive schizophrenia (TRS GWAS). The primary outcome was ACC Glu_corr._ For completeness, results from analyses with ACC Glx_corr_ as the outcome are reported. Glutamate metabolite estimates were z-scored and corrected for voxel tissue content. PRS R^2^ = variance in anterior cingulate cortex glutamate explained by the polygenic score only. SE = standard error. Raw p = raw p-value; p-value = competitive p-value, calculated using the synaptic background gene set.

**Supplementary Table 3.** Full regression output for the association between the NMDA receptor complex pathway polygenic score and glutamate concentrations in the anterior cingulate cortex

|  | **β** | **95% CI** | **p** |
| --- | --- | --- | --- |
| NMDAR-PGS | -0.25 | -0.49, -0.02 | 0.03 |
| Age | -0.02 | -0.05, 0.01 | 0.12 |
| Sex | -0.80 | -1.38, -0.21 | 0.009 |
| CPZE | 0.00 | 0.00, 0.00 | 0.63 |
| AP Response | 0.27 | -0.21, 0.74 | 0.26 |

NMDAR-PGS = NMDA receptor complex polygenic score; CPZE = chlorpromazine equivalent dose; AP Response = antipsychotic response (good versus. poor). The independent variable was anterior cingulate cortex glutamate, corrected for voxel tissue content and z-scored to control for site scanner effects. The first five genetic principal components were also included in the model to control for effects of population stratification.

**2.3. Exploratory post-hoc analyses**

Previous analyses of the STRATA-1 ^1^H-MRS cohort found an association between sex and ACC Glu_corr_ (Egerton et al., 2021). Therefore, the finding of a significant association between sex and ACC Glu_corr_ in this sub-cohort is unsurprising. Given the relative magnitude of the effect compared to NMDAR-PGS, exploratory post-hoc analyses were performed to better understand the effect of sex on relationships between the NMDAR-PGS and ACC Glu_corr_. There was no significant difference in NMDAR-PGS between males and females (t = 0.29, p = 0.78), and there was no significant sex*NMDAR-PGS interaction (β = -0.40, 95% CI = -1.03, 0.23, p = 0.21).

Given the negative direction of effect in the primary model, relationships between the NMDAR-PGS and ACC Glu_corr_ within the antipsychotic responder and non-responder groups were explored Here, the primary model was rerun to include a *NMDAR-PGS x responder status* interaction term. Results found no significant interaction between the NMDAR-PGS and responder status (β = 0.10, 95% CI = -0.34, 0.54, p = 0.64), which suggests that the association between the NMDAR-PGS and ACC glutamate was consistent across both antipsychotic responders and non-responders. An exploratory analysis stratified by antipsychotic response status found that the direction of effect between the NMDAR-PGS and ACC glutamate was consistent between groups (responder [n = 35]: β = -0.43, 95% CI = -0.83, -0.03, p = 0.35; non-responder [n = 32]: β = -0.21, 95% CI = -0.60, 0.17, p = 0.26. While the association between the NMDAR-PGS and ACC glutamate was not significant in the non-responder group, the primary model of the combined cohort likely has more power given that the signals in both strata have a consistent direction of effect.

**2.4. Investigating potential collider bias of antipsychotic response**

A previous analysis found that ACC Glu_corr_ was associated with antipsychotic response in this cohort (Egerton et al., 2021). In the current study, the TR NMDA-PGS was significantly higher in the antipsychotic responder group compared to the non-responder group (t = 2.48, p = 0.01). Because antipsychotic response is associated with the TR NMDA-PGS and ACC Glu_corr_, it is possible that the main effect of an association between the TR NMDA-PGS and ACC Glu_corr_ may have been distorted by the inclusion of antipsychotic response as a covariate.

To explore this further, we re-ran a regression model testing the association between TR NMDA-PGS and ACC Glu_corr_ without adjusting for antipsychotic response. The association between the NMDA-PGS and ACC Glu_corr_ remained significant (supplementary Table 4), with the same negative direction of effect as reported in our main results. This suggests that our main finding of a negative association between the NMDAR-PGS and ACC Glu_corr_ is not significantly biased by associations between antipsychotic response and these two variables.

**Supplementary Table 5.** Full regression output for the association between the NMDA receptor complex pathway polygenic score and glutamate concentrations in the anterior cingulate cortex, not including antipsychotic response group as a covariate

|  | **β** | **95% CI** | **p** |
| --- | --- | --- | --- |
| NMDAR-PGS | -0.29 | -0.51, -0.07 | 0.01 |
| Age | -0.02 | -0.05, 0.01 | 0.13 |
| Sex | -0.79 | -1.37, -0.20 | 0.01 |
| CPZE | 0.00 | 0.00, 0.00 | 0.77 |

NMDAR-PGS = NMDA receptor complex polygenic score; CPZE = chlorpromazine equivalent dose. The independent variable was anterior cingulate cortex glutamate, corrected for voxel tissue content and z-scored to control for site scanner effects. The first five genetic principal components were also included in the model to control for effects of population stratification.

# References

Anderson CA, Pettersson FH, Clarke GM, Cardon LR, Morris AP, Zondervan KT. Data quality control in genetic case-control association studies. Nature protocols. 2010; 5(9):1564-73. doi:10.1038/nprot.2010.116

Chang CC, Chow CC, Tellier LC, Vattikuti S, Purcell SM, Lee JJ. Second-generation PLINK: rising to the challenge of larger and richer datasets. Gigascience. 2015; 4(1):s13742-015. doi:10.1186/s13742-015-0047-8

Choi SW, Mak TSH, O’Reilly PF. Tutorial: a guide to performing polygenic risk score analyses. Nature Protocols. 2020;*15*(9), 2759-2772. doi:10.1038/s41596-020-0353-1

Das S, Forer L, Schönherr S, Sidore C, Locke AE, Kwong A, et al. Next-generation genotype imputation service and methods. Nature genetics. 2016; 48(10):1284-7. doi:10.1038/ng.3656

Egerton A, Murphy A, Donocik J, Anton A, Barker GJ, Collier T, et al. Dopamine and Glutamate in Antipsychotic-Responsive Compared With Antipsychotic-Nonresponsive Psychosis: A Multicenter Positron Emission Tomography and Magnetic Resonance Spectroscopy Study (STRATA). Schizophr Bull. 2021;47(2), 505-516. doi:10.1093/schbul/sbaa128

Hill WG. SEWALL WRIGHT's``Systems of Mating''. Genetics, 1996;*143*(4), 1499. PMCID: PMC1207415

Koopmans F, van Nierop P, Andres-Alonso M, Byrnes A, Cijsouw T, Coba MP, et al. SynGO: an evidence-based, expert-curated knowledge base for the synapse. *Neuron*. 2019;*103*(2), 217-234. doi:10.1016/j.neuron.2019.05.002

Loh PR, Danecek P, Palamara PF, Fuchsberger C, Reshef YA, Finucane HK, et al. Reference-based phasing using the Haplotype Reference Consortium panel. Nature genetics. 2016;*48*(11), 1443-1448. doi:10.1038/ng.3679

Pardiñas A, Smart S, Willcocks I, Holmans P, Dennison C, Lynham A, et al. Interaction testing and polygenic risk scoring to estimate the contribution of common genetic variants to treatment-resistant schizophrenia. *JAMA Psychiatry.* 2021. doi:10.1001/jamapsychiatry.2021.3799

Pardiñas AF, Holmans P, Pocklington AJ, Escott-Price V, Ripke S, Carrera N, et al. Common schizophrenia alleles are enriched in mutation-intolerant genes and in regions under strong background selection. Nature genetics. 2018; 50(3):381-9. doi:10.1038/s41588-018-0059-2

Purcell S, Neale B, Todd-Brown K, Thomas L, Ferreira MA, Bender D, et al. PLINK: a tool set for whole-genome association and population-based linkage analyses. The American journal of human genetics. 2007; 81(3):559-75. doi: 10.1086/519795

Stanaway IB, Hall TO, Rosenthal EA, Palmer M, Naranbhai V, Knevel R, et al. The eMERGE genotype set of 83,717 subjects imputed to~ 40 million variants genome wide and association with the herpes zoster medical record phenotype. Genetic epidemiology.  2019;43(1), 63-81. doi:10.1002/gepi.22167

Thul PJ, Lindskog C. The human protein atlas: a spatial map of the human proteome. Protein Science. 2018;27(1), 233-244. doi:10.1002/pro.3307

# Appendix I

**List of genes included in each NMDA receptor gene set**

| GOBP Positive Regulation of NMDA Glutamate Receptor Activity |
| --- |
| *CCL2 CCR2 EPHB2 IFNG PINK1 RGS9* |
| GOCC NMDA Selective Glutamate Receptor Complex |
| *GRIN1 GRIN2A GRIN2B GRIN2C GRIN2D GRIN3A GRIN3B NLGN1 PTK2B SHANK1* |

GOBP = Gene Ontology Biological Process, GOCC = Gene Ontology Cellular Component
